# Polariton condensation in an organic microcavity utilising a hybrid metal-DBR mirror

**DOI:** 10.1038/s41598-021-00203-y

**Published:** 2021-10-22

**Authors:** Kirsty E. McGhee, Anton Putintsev, Rahul Jayaprakash, Kyriacos Georgiou, Mary E. O’Kane, Rachel C. Kilbride, Elena J. Cassella, Marco Cavazzini, Denis A. Sannikov, Pavlos G. Lagoudakis, David G. Lidzey

**Affiliations:** 1 Department of Physics and Astronomy, University of Sheffield, Hicks Building, Hounsfield Road, Sheffield, S3 7RH UK; 2 Centre of Photonics and Quantum Materials, Skolkovo Institute of Science and Technology, Moscow, Russian Federation 121205; 3 Department of Physics, University of Cyprus, P.O. Box 20537, 1678 Nicosia, Cyprus; 4 Consiglio Nazionale delle Ricerche, Istituto di Scienze e Tecnologie Chimiche “Giulio Natta”, Via C. Golgi 19, 20133 Milano, Italy; 5 Department of Physics and Astronomy, University of Southampton, University Road, Southampton, SO17 1BJ UK

**Keywords:** Materials science, Materials for devices, Materials for optics, Optics and photonics, Lasers, LEDs and light sources, Optical materials and structures

## Abstract

We have developed a simplified approach to fabricate high-reflectivity mirrors suitable for applications in a strongly-coupled organic-semiconductor microcavity. Such mirrors are based on a small number of quarter-wave dielectric pairs deposited on top of a thick silver film that combine high reflectivity and broad reflectivity bandwidth. Using this approach, we construct a microcavity containing the molecular dye BODIPY-Br in which the bottom cavity mirror is composed of a silver layer coated by a SiO_2_ and a Nb_2_O_5_ film, and show that this cavity undergoes polariton condensation at a similar threshold to that of a control cavity whose bottom mirror consists of ten quarter-wave dielectric pairs. We observe, however, that the roughness of the hybrid mirror—caused by limited adhesion between the silver and the dielectric pair—apparently prevents complete collapse of the population to the ground polariton state above the condensation threshold.

## Introduction

A cavity-polariton is a quasiparticle formed by coupling an exciton to a confined photon mode within an optical cavity. Cavity-polaritons are bosons and have an effective mass of ~ 10^−11^ that of an atom^1^ and therefore undergo Bose–Einstein condensation (BEC) at temperatures much higher than those observed with atomic gases^2^. In this process, polaritons condense into a single state once their density reaches a certain critical threshold, with all polaritons within the condensate having the same energy, momentum, and phase. Photons emitted by the condensate share these properties and as such possess the same characteristics as a laser^3^. This type of emission has been termed polariton lasing^4–7^, with these devices being of emerging interest as novel low-threshold lasers and light-sources.

In recent years, increasing attention has been shown to the exploration of polariton condensation effects in organic semiconductor microcavities^1,8–12^. Here, Frenkel excitons have a binding energy that is much greater than *k*_*B*_*T* at room temperature^13^ and thus organic exciton–polariton microcavities are able to undergo room temperature condensation^1,8,9^, a property that opens new routes to practical applications. A range of different organic semiconductors have been shown to undergo polariton condensation, including single crystals^8,14^, conjugated polymers^1,15,16^, and small-molecules, either in a crystalline form^9,17^ or dispersed in a transparent polymer-matrix^18–20^.

Many microcavities are based on two distributed Bragg reflectors (DBRs) placed either side of the active layer. Such DBRs generally consist of a stack of alternating high- and low- refractive index quarter-wave layers, producing mirrors that possess both high reflectivity (> 99%) and low optical absorption. In a Fabry-Pérot microcavity, the reflectivity of the cavity mirrors directly controls the degree of optical confinement and the cavity mode linewidth. Optical confinement can be defined by the cavity quality-factor (*Q-*factor) that is expressed using *Q* = λ/Δλ, where λ is the cavity mode wavelength and Δλ is its spectral full-width at half maximum (FWHM) ^21^.

Many organic semiconductors have been shown to enter the strong coupling regime using microcavities with relatively low-reflectivity mirrors. Despite the fact that photon mode linewidths in low *Q*-factor cavities are relatively large (10–100 meV), the high oscillator strength of organic semiconductors results in large Rabi splitting energies (the energy splitting between the upper and lower polariton branches at resonance), $\hslash {\Omega }_{Rabi}$, of 100 s meV to ~ 1 eV ^22–25^. Many of the highest Rabi splittings using organic semiconductors have been observed in structures based on two metallic reflectors (at least one of which is semi-transparent) with *Q*-factors of 10–65 ^22,26–29^. Here, the small optical penetration depth of metals such as silver (Ag) results in a high degree of optical confinement, which reduces the effective cavity length (*L*_eff_). As the Rabi splitting is inversely proportional to *L*_eff_, ‘all metal’ cavities are generally characterised by increased Rabi splittings, and—as they are simple to fabricate via thermal evaporation—have effectively become the structure of choice in which to explore polaritonic properties that do not rely on non-linear effects^10,26,27^.

To achieve polariton condensation, however, relatively extended polariton lifetimes are required. Most exciton–polariton microcavities that have been reported to undergo condensation are based on two DBR mirrors and typically have *Q*-factors of several hundred to > 1000 ^19,30,31^. (It is worth noting here that Ren et al*.* recently demonstrated polariton condensation in a single crystal all-metal cavity^14^; however, it is difficult to fabricate such single crystals into microcavities with sufficiently high uniformity to support polariton condensation.) The reflectivity of a DBR is a strong function of the number of dielectric pairs^21^, and thus such mirrors often comprise in excess of 30 individual layers^1,18,32,33^. Fabricating such structures is time-consuming, costly, and prone to error.

In this study, we address this issue and explore a new type of ‘hybrid’ dielectric mirror that is composed of a thick layer of a metal (Ag) onto which we deposit one or more dielectric mirror pairs. Here, the function of the silver mirror is to provide a high, broadband optical reflectivity that is then enhanced by a low number of DBR pairs, with such structures being significantly easier to fabricate. We characterise the properties of these hybrid mirrors and show that a structure composed of 4 DBR pairs backed by a silver film have a comparable reflectivity to that of a conventional 10-pair DBR. We explore the properties of strongly-coupled cavities made using a hybrid mirror, an organic semiconductor dye, and a conventional DBR, and show that cavities incorporating a 1-pair hybrid mirror have a Rabi splitting energy that is around 14% larger than a 9-pair/10-pair DBR–DBR control. We also demonstrate enhanced confinement, with structures based on a 10-pair hybrid mirror having a *Q*-factor that is 24% larger than the DBR control. We then explore the non-linear properties of a cavity utilising a 1-pair hybrid mirror and show that it can undergo polariton-condensation. However, we find in contrast to the DBR control cavity, emission from the hybrid cavity does not fully coincide with the bottom of the lower polariton branch. Using atomic force microscopy (AFM) and scanning electron microscopy (SEM), we show that this effect most likely results from disorder at the interface between the Ag mirror and the DBR.

## Results

### Hybrid distributed Bragg reflectors (DBRs)

**Figure 1:**
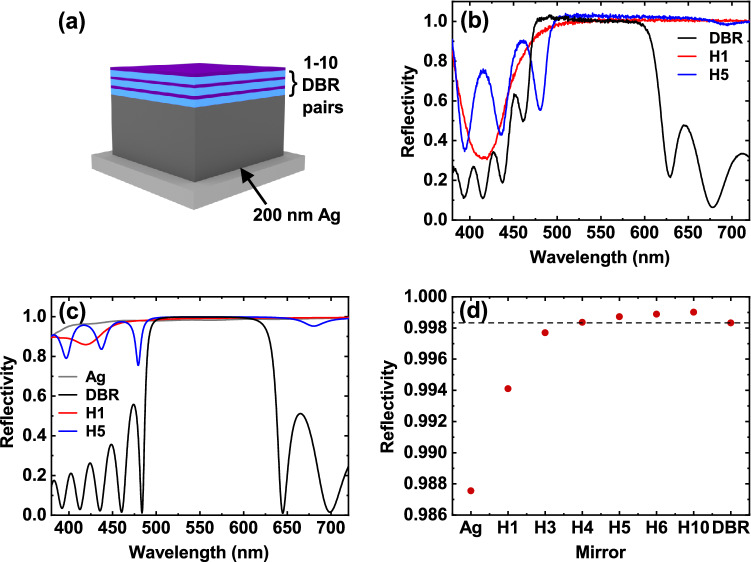
Hybrid metal-DBR mirrors. (**a**) Schematic of hybrid mirror. (**b**) Shows the experimentally measured reflectivity of the 1-pair hybrid (red), 5-pair hybrid (blue) and 10-pair DBR (black) mirrors, with transfer matrix reflectivity data for the same mirrors plus Ag (grey) shown in (**c**). (**d**) Plots the calculated reflectivity of each mirror at 550 nm. The horizontal dashed black line corresponds to the reflectivity of a 10-pair DBR. In (**b**–**d**), legend and x-axis labels ‘H*X*’ correspond to the hybrid mirrors, where *X* is the number of DBR pairs on top of the Ag, and ‘Ag’ and ‘DBR’ correspond to an Ag film and the 10-pair DBR, respectively.

We have fabricated hybrid metal-DBR mirrors by thermally evaporating a 200 nm Ag film onto a quartz-coated glass substrate, followed by the deposition of various numbers (between 1 and 10) of SiO_2_/Nb_2_O_5_ quarter-wave pairs (refractive index ~ 1.46 and ~ 2.20, respectively) by electron beam evaporation. The structure of such a hybrid mirror is shown schematically in Fig. 1a. Figure 1b shows the normal incidence white light reflectivity of a 1-pair hybrid, 5-pair hybrid and 10-pair (conventional) DBR mirror, with a 200 nm Ag mirror used as a reference. Figure 1c plots the reflectivity determined using a transfer matrix reflectivity (TMR) model for the same three mirrors and also that of silver. Here, our structures are based on a relatively thick silver film (200 nm) to maximise its optical reflectivity. Our TMR model (see Figure S1 in the “Supplementary Information”) indicates that the reflectivity of a silver film does not increase once its thickness exceeds 200 nm. The cavity structures that we use (see "Optical properties of microcavities fabricated using hybrid-DBR mirrors'' below) are designed to extract light through a conventional ‘top’ DBR, and thus our approach allows us to use relatively thick silver films having high reflectivity and low optical transmission on the bottom mirror.

We can see from Fig. 1c that the hybrid mirrors have a comparable reflectivity to a conventional DBR, together with a significantly broader stopband (high-reflectivity region) resulting from the use of a silver mirror. Indeed, our TMR simulations indicate that the hybrid mirrors (except for the 1-pair hybrid) have a reflectivity that is > 99.5% over a bandwidth of 130–140 nm (corresponding to ~ 530 meV). This high-reflectivity band is wider than the stop-band of the control DBR, which is limited to around 90 nm (~ 350 meV). This widened stop-band is potentially useful, as the large oscillator strength of organic semiconductors can result in large Rabi splittings (100’s of meV) that are often greater than the width of the stopband of a conventional DBR. Figure 1d plots the TMR model reflectivity of each mirror explored (Ag, hybrid, DBR) at 550 nm. It can be seen from this that, at this wavelength, hybrid mirrors with five or more pairs have increased reflectivity compared to a 10-pair DBR.

### Optical properties of microcavities fabricated using hybrid-DBR mirrors

We have used the hybrid mirrors outlined in Fig. 1 to fabricate a series of strongly-coupled microcavities containing the molecular dye bromine-substituted boron-dipyrromethene (BODIPY-Br). This dye combines high oscillator strength and relatively high photoluminescence quantum yield, and has previously been shown to undergo polariton-condensation^18,34^. Additionally, its photoluminescence (PL) emission lies in a spectral region not easily accessible with traditional inorganic semiconductors (green/yellow), making it desirable for laser applications.

**Figure 2:**
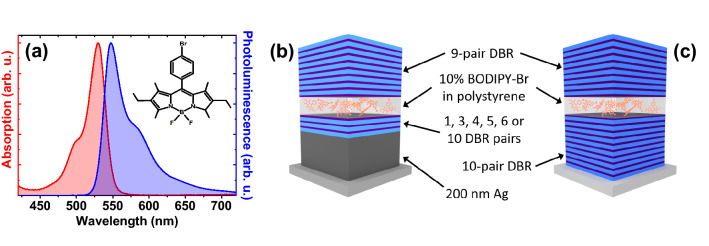
(**a**) Normalised absorption (red) and photoluminescence (blue) spectra of BODIPY-Br at 10% by mass in polystyrene. The molecular structure is shown in the inset. Schematic of (**b**) the hybrid Ag-DBR microcavity and (**c**) a conventional DBR–DBR microcavity.

To process BODIPY-Br it was first dispersed into a polystyrene (PS) matrix at 10% by mass in toluene. Here, the function of polystyrene is to prevent molecular aggregation, a process that results in the quenching of BODIPY-Br luminescence. To create thin films, the BODIPY-Br/PS blend was deposited by spin-coating. Figure 2a shows the normalised absorption and PL spectra of a BODIPY-Br/PS film on a quartz substrate, with the molecular structure of BODIPY-Br shown in the inset. It can be seen that the absorption and PL peak around 530 nm and 547 nm, respectively.

Microcavities were fabricated by spin-coating a BODIPY-Br/PS solution onto the hybrid-DBR mirrors. This was followed by the deposition of a 9-pair DBR (via e-beam evaporation) on top of the active layer to create the full structure as shown in Fig. 2b. In all cases, the thickness of the BODIPY-Br/PS layer was chosen to give a cavity mode positioned around 547 nm. As a control, we also fabricated a DBR–DBR cavity with a 10-pair bottom DBR, an active BODIPY-Br/PS layer, and a 9-pair top DBR. The structure of this cavity is shown in Fig. 2c.

**Figure 3:**
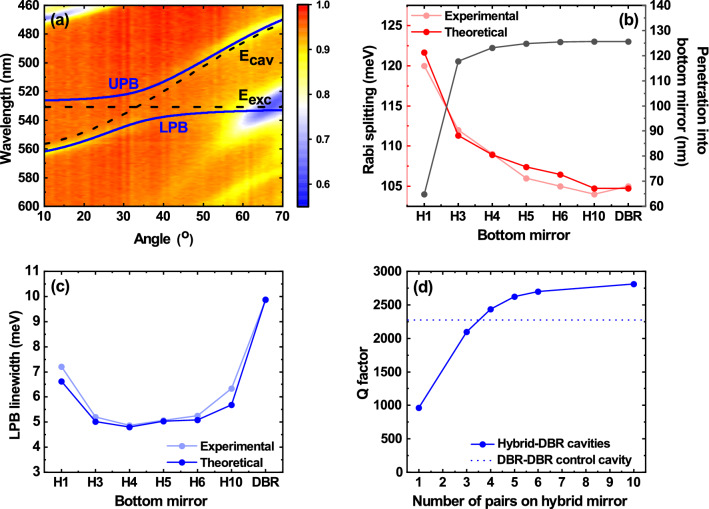
The effect of the number of hybrid mirror pairs on microcavity optical properties. (**a**) Plots the angle-dependent reflectivity measurement of a 4-pair hybrid mirror cavity. Fits of upper (UPB) and lower (LPB) polariton branches (blue solid lines) are made using a standard two-level oscillator model. The dispersion of the cavity photon mode (E_cav_) and the exciton energy (E_exc_) are also shown (black dashed lines). (**b**) Shows the Rabi splitting for the hybrid mirror cavities and the DBR control, where good agreement can be seen between experimental (light red, two-level oscillator) and theoretical (dark red, TMR) values. The modelled penetration of the electromagnetic field into the bottom mirror (grey) is also shown here. (**c**) Shows the linewidth of the LPB for the hybrid mirror and DBR cavities, comparing experimental (light blue) and theoretical (dark blue, from TMR) values. In both (**b**) and (**c**), the x-axis labels ‘H*X*’ correspond to the hybrid mirrors, where *X* is the number of DBR pairs on top of the Ag, and ‘DBR’ corresponds to the 10-pair DBR. In (**d**), we plot the modelled *Q*-factor for hybrid mirror cavities with cavity mode at 547 nm. The modelled *Q*-factor of the DBR control cavity is indicated by a horizontal blue dotted line.

To characterise the cavities, we first performed angle-dependent white light reflectivity measurements to confirm they operated within the strong coupling regime. Here, measurements were performed using a goniometer setup in which a lens system positioned on a rotating optical rail was used to focus unpolarised white light onto the cavity surface. A series of lenses on a second rotating optical rail were used to collect the light and deliver it to a spectrometer (see Ref.^35^ for more details). The angle-dependent reflectivity of a 4-pair hybrid cavity is shown in Fig. 3a. Here we observe two dispersive dips in the reflectivity spectra that undergo anticrossing around the energy of the BODIPY-Br exciton (530 nm). These two features are identified as the lower (LPB) and upper polariton (UPB) branches, and we have used a two-level oscillator model to fit their dispersions (the solid lines in Fig. 3a). It can be seen that the description of the measured data is excellent.

We have used our model to extract the Rabi splitting energy for each of the different cavities and this is shown in Fig. 3b together with experimentally measured data. We note that the Rabi splitting energy is primarily dependent on the number of absorbers per unit length in a cavity as discussed Sect. 2 of the “Supplementary Information”. However, here we attribute changes in the Rabi splitting to variations in the effective cavity length (*L*_*eff*_). As can be seen, we observe an increase in Rabi splitting energy as the number of DBR pairs on the bottom hybrid mirror is reduced, with the cavity incorporating a 1-pair hybrid DBR having a Rabi splitting energy that is around 14% larger than the DBR–DBR control. Evidently, as the number of DBR pairs in the hybrid mirror is reduced, *L*_*eff*_ is reduced which results in an effective “concentration” of the electromagnetic field into the active cavity region and an increase in the Rabi splitting. In Figure S3 of the “Supplementary Information”, we show electric field simulations of the cavity mode for the 1-pair and 5-pair hybrid cavities and the DBR control cavity, showing the penetration of the field into the different mirrors and the refractive index variation in each structure. This confirms the increased effective cavity length of structures incorporating an increased number of DBR pairs and we have calculated this (see Figure S2 of the “Supplementary Information”) using Ref.^36^. However, for simplicity, in Fig. 3b we show the penetration depth of the electromagnetic field into the bottom mirror only.

To explore the effect of the hybrid mirror on cavity *Q*-factor, we focused imaged a 445 nm pulsed laser beam into a 4 μm diameter spot on the cavity surface to generate PL emission. A *k*-space imaging setup was then used to collect the luminescence and image it into a CCD spectrometer, allowing the FWHM emission linewidth of the LPB at normal incidence to be determined. Figure 3c plots the normal incidence LPB linewidth measured from the various structures. It can be seen that there is an apparent reduction in LPB linewidth as the number of mirror pairs is increased, with this trend reversing for structures having more than 5 DBR pairs. However, this ‘reversal’ effect largely results from the fact that there was a small variation in the cavity mode wavelength caused by unintended variations in the thickness of the cavity active layer caused by the spin-coating process. This modifies the relative exciton–photon fraction of the LPB, with the photon fraction increasing (and linewidth reducing) as the negative detuning (energetic separation between the photon mode and the exciton) increases. We can account for this effect in our model by using the known *n* and *k* data for the BODIPY-Br/PS film. This was input into a TMR model to describe the dispersion of the LPB and UPB for each cavity. From this, we were able to calculate the physical thickness of the organic film, the position of the cavity mode at normal incidence, and the LPB linewidth. This data is also plotted in Fig. 3c, where we again find a good agreement between model and experimental results.

Using our model, we are then able to calculate expected cavity *Q*-factor as a function of the number of DBR mirror pairs. Here, we ‘turn-off’ the exciton oscillator strength and calculate the LPB linewidth and cavity *Q*-factor assuming in all cases the cavity mode is located at 547 nm. The results of our model are shown in Fig. 3d. Here, we find the cavity *Q*-factor increases as the number of DBR pairs in the hybrid mirror is increased, and for 4 or more pairs, exceeds that of the DBR–DBR control. Furthermore, our model suggests that a cavity utilising a hybrid mirror consisting of 10 DBR pairs on top of an Ag film is expected to have a *Q*-factor that is 24% larger than an equivalent ‘conventional’ cavity whose bottom mirror does not contain Ag, (i.e. simply composed of a 10-pair DBR).

As expected, we find that the measured and modelled polariton linewidths shown in Fig. 3c are strongly dependent on mixing between the exciton and photon. For example, for the 1-pair hybrid cavity, we determine (by modelling and experiment) an LPB linewidth of around 7 meV at normal incidence. However, the *Q*-factor of the same cavity is ~ 1000, suggesting an uncoupled photon-linewidth of around 2.2 meV. This substantial broadening of the LPB linewidth occurs even though the excitonic fraction of this state is relatively small (being 13% exciton and 87% photon). As we discuss below, this broadened polariton linewidth masks a small amount of site-to-site variation of the cavity photon energy that occurs as a result of structural disorder within the cavity that only becomes apparent when polaritons undergo condensation and lasing.

### Polariton condensation in hybrid-DBR microcavities

We have explored our structures for evidence of polariton condensation. Here, it was decided to explore such effects in cavities that had the largest difference in their structural properties. Condensation effects were therefore studied in a 1-pair hybrid cavity and compared to a DBR–DBR control. In these experiments, the hybrid cavity utilised an 8-pair top DBR mirror. The DBR–DBR control structure was based on 10 and 8 DBR pairs, with the 10-pair mirror being fabricated by Helia Photonics Ltd. In both cases, the cavities had a very similar exciton–photon detuning, with the LPB being positioned at 567 and 569 nm in the hybrid and DBR cavities, respectively.

To generate polariton condensation, the microcavities were pumped non-resonantly at normal-incidence using a single-shot imaging technique in reflection configuration, with emission detected using *k-*space imaging. Here, the laser pulses (width of 150 fs, repetition frequency of 15 Hz) were focused onto the cavity surface into a spot with a FWHM diameter of 30 μm. For both cavities, the excitation laser wavelength was tuned to the first Bragg minimum at the edge of the stopband (467 nm for the hybrid cavity and 462 nm in the DBR–DBR control) to maximise the transmission of light into the cavity. In the data presented below, the PL intensity for the power dependence graphs was obtained by integrating single-shot real-space images of the cavity emission. The blueshift and FWHM data were extracted from the single-shot *k*-space dispersion images profiles filtered over ± 0.075 cm^−1^ range around *k* = 0.

**Figure 4:**
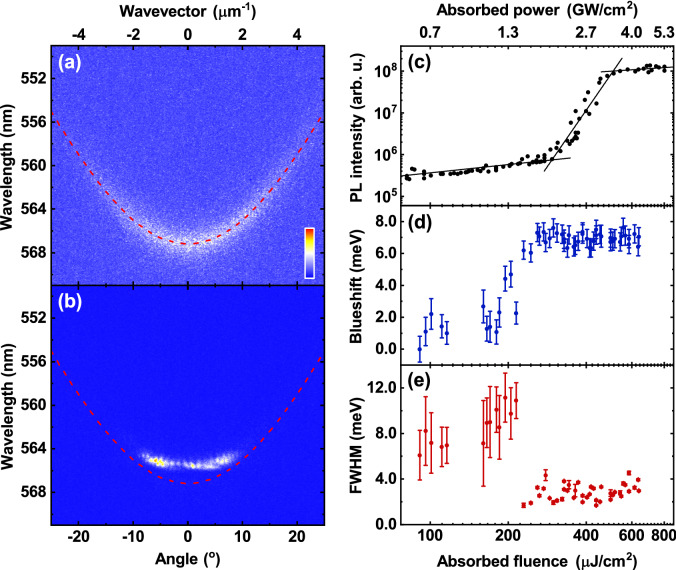
1-pair hybrid mirror cavity condensation results. Normalised dispersions taken (**a**) below threshold and (**b**) above threshold. In both cases the red dashed line is a fit to the LPB in the linear regime. (**c**–**e**) Show the dependence of (**c**) PL intensity, (**d**) blueshift, and (**e**) full-width at half-maximum (FWHM) of the LPB on the absorbed pump fluence. From straight line fits to the power-dependent PL intensity in (**c**) (black solid lines), we determine a condensation threshold for this cavity of 295 μJ/cm^2^. Here we see a non-linear increase in PL intensity, a spectral blueshift, and a decrease in the FWHM of the LPB. The error bars in (**d**) and (**e**) were calculated from the error on a Gaussian fit to the LPB at each fluence.

Figure 4a plots the normalised PL emission from the hybrid mirror cavity recorded below the condensation threshold, together with a TMR model fit to the LPB dispersion. Here it can be seen that emission is initially distributed across a large angular range. We find that as the pump fluence is increased above the condensation threshold, there is a non-linear increase in PL intensity (see Fig. 4c), accompanied by an energy blueshift (Fig. 4d) and a decrease in the linewidth (Fig. 4e). Such blueshifts of the LPB have previously been explained on the basis of a partial saturation of the BODIPY optical transition^12^. Figure 4b shows the normalised PL emission above threshold, with the fit to the LPB below threshold also shown, allowing the extent of the energy blueshift to be clearly seen. Specifically, we determine a condensation threshold (determined from the measured absorbed fluence) of 295 μJ/cm^2^ which is accompanied by a blueshift of 7 meV and a reduction in LPB FWHM linewidth from 11 meV to ~ 2.8 meV.

**Figure 5:**
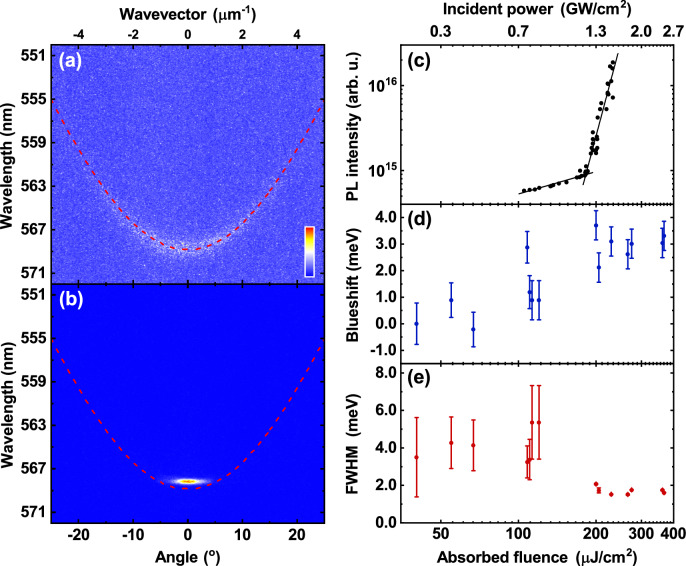
DBR–DBR control cavity condensation results. Normalised dispersions taken (**a**) below threshold and (**b**) above threshold. In both cases the red dashed line is a fit to the LPB in the linear regime. (**c**–**e**) Show the dependence of (**c**) PL intensity, (**d**) blueshift, and (**e**) full-width at half-maximum (FWHM) of the LPB on the absorbed pump fluence. From straight line fits to the power-dependent PL intensity in (**c**) (black solid lines), we determine a condensation threshold for this cavity of 180 μJ/cm^2^. Here we see a non-linear increase in PL intensity, a spectral blueshift, and a decrease in the FWHM of the LPB. The error bars in (**d**) and (**e**) were calculated from the error on a Gaussian fit to the LPB at each fluence.

The equivalent condensation dataset to Fig. 4 for the DBR–DBR control is shown in Fig. 5. From part (c), we determine a condensation threshold of 180 μJ/cm^2^, which is accompanied by a blueshift of approximately 3 meV (part d) and a reduction in emission linewidth from 5 meV to ~ 1.6 meV (part e). Interestingly, it is clear that the blueshift observed in the hybrid mirror cavity (7 meV) is more than twice that in the DBR cavity (3 meV). In Figure S4 in the “Supplementary Information”, we use a TMR model to show that a larger blueshift is expected in the hybrid mirror cavity for a given reduction in molecular oscillator strength, an effect that we attribute to its enhanced Rabi splitting^37^.

If we compare the distribution of emission in *k*-space for both cavities above threshold (Figs. 4b and 5b), it is evident that in the DBR–DBR control, the emission is strongly concentrated around the bottom of the LPB. In contrast, we do not evidence a full collapse to *k* = 0 in the hybrid mirror cavity; rather, the emission appears to be distributed over a wider range of *k*-vectors and energies and is apparently finely structured. We investigate the cause of this effect in "Investigating cavity homogeneity" below. For completeness, we also plot real-space images of the emission from both types of cavity below and above condensation threshold in Figure S5. Below threshold, the emission from both cavities is unstructured and has a size approximating the laser excitation spot (20–30 μm (FWHM)). Above threshold, the condensate in both types of cavity has a complex structure and consists of one or more separate domains having a size of between ~ 5 and 20 μm. This effect has been previously observed and has been ascribed to interactions between the polariton condensate and the exciton reservoir^38,39^.

### Investigating cavity homogeneity

To explore the origin of the structure in the LPB dispersion above threshold, we used a spatial mapping technique to measure the PL emission from the LPB as a function of position for both the 1-pair hybrid cavity and the DBR–DBR control. Here, the sample was mounted on a motorised (*x*, *y*) stage that was incorporated into the *k*-space imaging setup, with sub-threshold emission mapped over an area of (26 × 26) μm^2^ in step-sizes of 1 μm using a laser focused to a diameter of around 4 μm. From the PL spectra recorded, the peak emission energy at *k* = 0 at each position was then determined across the cavity surface. We also calculated the range of peak emission energies across the cavity and their standard deviation (SD). To gauge the significance of our results, we also attempted to characterise the spectral resolution of the mapping system. Here, light from a Ne/Ar lamp was directed into the spectrometer with its entrance slit set to 20 μm, and it was found that the emission line at 585 nm had an apparent linewidth (FWHM) of 0.68 meV (see Figure S6). Note, however, this measurement should only be viewed as an approximation of the true system resolution, as the *k*-space mapping setup required the entrance slit of the spectrometer to be fully-open in order to characterise the emission dispersion.

**Figure 6:**
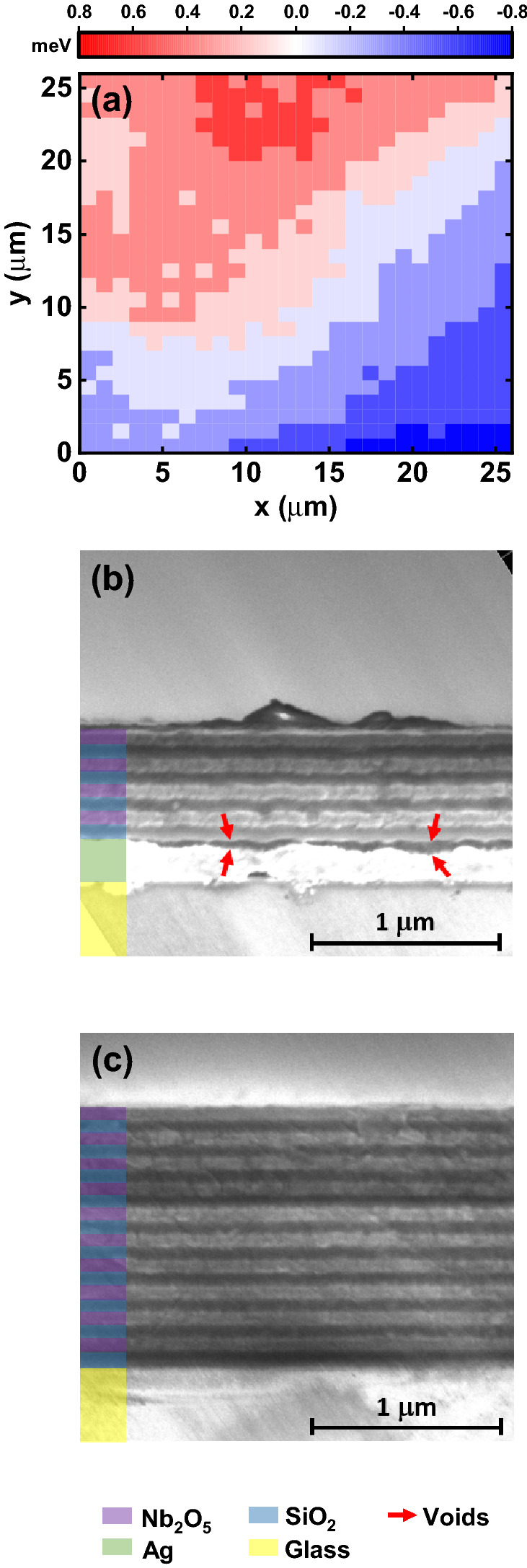
(**a**) Shows a sub-threshold polariton emission map of the variation in LPB emission energy from the DBR–DBR cavity from which condensation was observed. The colour scale corresponds to the variation of the LPB energy (in units of meV) around its average emission energy. (**b**,**c**) Show SEM images of the 4-pair hybrid and 10-pair DBR mirrors respectively. In (**b**), voids can be seen between the Ag and first SiO_2_ layer.

We first explored the emission from the DBR–DBR control cavity. A typical (25 × 25) μm^2^ image is shown in Fig. 6a, with a second PL map shown in Figure S6 in the “Supplementary Information”. From Fig. 6a it can be seen that there is a gradual transition in LPB energy across the image, indicating that there is a slight ‘wedge’ in the cavity optical path-length. This effect possibly results from a gradual variation in the thickness of the BODIPY-Br active layer. From an analysis of the two images collected, it was determined that the peak energy of the LPB is distributed over an energy range of around 1.3 meV with a standard deviation of 0.29 meV. For completeness, Figure S6 also plots example spectra collected at two points, approximately corresponding to the extremes of LPB energy. Here, each peak has a linewidth of 4 meV, with the peaks differing in the peak emission energy by ~ 1 meV, with this difference being greater than the spectral resolution of our spectrometer.

Using the same technique, we attempted to characterise the 1-pair hybrid cavity. Here, the cavity that was used had a slightly lower *Q*-factor than the cavity discussed in "Optical properties of microcavities fabricated using hybrid-DBR mirrors" as a result of a reduced number (8) of DBR pairs in the top mirror. In this case, due to the relatively broad LPB linewidth (10 meV at *k* = 0) we were unable to resolve statistically significant differences in LPB energy across the cavity surface.

Therefore, to further characterise cavity homogeneity, cross-sectional SEM images were recorded, with AFM and profilometry maps also made to explore the structure of the different cavity mirrors. Figure 6b,c show SEM images of the 4-pair hybrid and 10-pair DBR mirrors, respectively. Significantly, we find evidence for voids between the Ag and SiO_2_ layer in the hybrid mirror structure having a lateral length-scale of a few 100 nm, with resultant disorder apparently propagating into the DBR layers. In contrast, no such voids are evident in the DBR–DBR control, with the individual mirror layers having a high degree of uniformity. We speculate that such voids in the hybrid mirror cavity are highly likely to result in local fluctuations in effective cavity length. We have also used AFM to image the various layers that constitute the hybrid mirror and the DBR control and have used this to extract the root mean square roughness (see Table 1 and images in Figure S7). Here, we find that both the Ag and SiO_2_ layers are individually very smooth; however, we detect enhanced roughness from both an Ag/SiO_2_ bilayer and the 1-pair hybrid mirror. Such layers are in fact rougher than the 10-pair DBR fabricated by Helia Photonics Ltd. We suspect the enhanced roughness of the 1-pair hybrid mirror results from poor adhesion between the Ag and SiO_2_ layers and results in the voids observed in Fig. 6b. These results are further supported by surface profilometry measurements, which we discuss in Sect. 6 of the “Supplementary Information”.

**Table 1:**
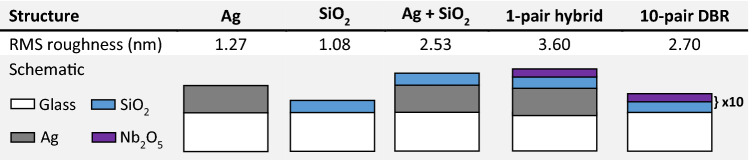
Root mean square (RMS) roughness of the different structures investigated, with a schematic of the structure shown beneath.

These values were obtained by averaging multiple AFM maps.

## Discussion

We have observed polariton condensation in a hybrid mirror cavity and in the DBR–DBR control at an absorbed fluence threshold of 295 μJ/cm^2^ and 180 μJ/cm^2^, respectively. It appears that the ratio of condensation thresholds scales inversely with cavity *Q*-factor, with the DBR–DBR cavity undergoing condensation at a threshold that is 1.6 times lower than that of the hybrid mirror cavity and possessing a cavity *Q*-factor that is 1.7 times greater (1190 and 710, respectively). We note that the condensation threshold of 180 μJ/cm^2^ in the DBR–DBR control observed here is slightly lower than that observed in previous studies on a similar microcavity containing BODIPY-Br which was determined as 530 μJ/cm^2^^18^. We suspect such differences may result from the shorter pump-laser pulse-lengths used here (150 fs compared to 2 ps pulses used in previous experiments), with reductions in lasing threshold being previously observed in weakly-coupled organic microcavities excited using shorter pump-pulses^40^.

We now discuss polariton relaxation processes in the two types of cavities explored. It is clear that the mapping experiments described in "Investigating cavity homogeneity" evidence a slowly changing energy landscape in the DBR–DBR cavity, with the lateral size of the polariton condensate being either similar to, or smaller than the length-scales over which the cavity path-length fluctuates. Specifically, we find that the energy of the LPB changes by around 0.6 meV over distances of around 10–20 μm (the typical condensate size). This level of disorder is smaller than the linewidth of the condensate emission (1.6 meV at* k* = 0), and thus the ‘energetically flat’ landscape of this cavity allows complete relaxation to the ground polariton state.

In the hybrid mirror cavity, however, we find that condensate emission does not undergo complete collapse to *k* = 0, but is finely structured and distributed over a wider range of *k*-vectors. In these cavities, SEM images evidence spatial disorder at the interface between the DBR and the silver mirror having a lateral length-scale of a few 100 nm, with this disorder propagating through the DBR. At present, the relatively large linewidth (10 meV) of the LPB below the condensation threshold (which is mainly broadened by exciton–photon mixing) effectively masks energetic disorder within the cavity. Once the condensation threshold is reached, however, the narrowed condensate emission linewidth now allows energetic disorder within the cavity to be evidenced through the fine-structure that emerges in the emission dispersion curve (see Fig. 4b). This structure indicates that the ‘fragmented’ condensates that are formed exist in a relatively disordered landscape resulting from local variations in the cavity path-length, with the condensate linewidth (2.8 meV at *k* = 0) being almost a factor of two greater than that observed in the DBR–DBR control cavity. We note that similar structure in *k-*space emission has previously been observed in GaN and MeLPPP microcavities and has also been attributed to thickness variations in the active layers^41,42^.

We suspect that the increased structural disorder in the hybrid mirror cavity results from poor adhesion between the Ag and SiO_2_ layers, which results in a series of voids at this interface. We have made preliminary attempts to reduce this issue; for example, we have determined that the surface roughness of an Ag/SiO_2_ bilayer is not dependent on the deposition rate of SiO_2_ (see Sect. 5 of the “Supplementary Information”). It is possible that other techniques could be used to improve the quality of the interface between these layers—for example, using adhesion promoters or thermal annealing during deposition to encourage the SiO_2_ to better adhere to the Ag film. Conversely, it may be possible to construct ‘upside-down’ hybrid mirror cavities, with the Ag layer deposited onto the final top surface of the cavity. This approach might result in an improved interface between the SiO_2_ and Ag layers; however, such cavities would likely require optical measurements to be made through the bottom glass substrate. While this would not be an issue for linear optical spectroscopy, such a geometry is not well suited to transient measurements due to multiple internal reflections within the thick glass substrate.

## Summary

We have developed a strategy to realise high-reflectivity ‘hybrid’ mirrors based on a silver film coated with a series of quarter-wave DBR pairs. This approach allows very high-reflectivity mirrors to be realised that are considerably simpler to fabricate than a regular dielectric mirror. Such mirrors are also anticipated to have enhanced thermal conductivity compared to a regular DBR and thus may dissipate heat more effectively when they are pumped with high energy lasers. We use such mirrors to fabricate a series of strongly-coupled organic semiconductor microcavities and show that by using a silver film coated with 4, 5 or 6 DBR mirror pairs, we create a structure having both a higher *Q*-factor and a larger Rabi splitting than could be realised using a conventional cavity based on two regular 9- and 10-pair dielectric mirrors. We use this approach to fabricate a strongly-coupled cavity in which the bottom hybrid mirror consists of a layer of silver coated by a single DBR pair, and show that this structure is able to demonstrate polariton condensation with a slightly increased threshold compared to a conventional DBR–DBR control cavity. Interestingly, however, we evidence a partial break-up of the *k*-space emission above threshold in the hybrid mirror cavity that we conclude occurs from spatial disorder at the interface between the single DBR pair and the silver film which propagates through the structure. In contrast, very little disorder is evidenced in the DBR–DBR based cavity, a result confirmed by mapping the LPB emission energy across the cavity surface. Despite such disorder effects, we believe that the approach demonstrated here allows a simplified route to construct high-performance mirrors for organic polariton lasers.

## Methods

### Sample preparation

BODIPY-Br was dissolved at 10% by mass in a solution of 35 mg/mL PS (Sigma-Aldrich, molecular weight ~ 192,000) in toluene and spin-coated onto a quartz-coated glass substrate. A Bruker DektakXT profilometer was used to measure the thickness of the films. To fabricate the hybrid mirrors, a silver film was first evaporated onto a quartz-coated glass substrate using an Ångstrom Engineering thermal evaporator. The DBR mirror pairs were fabricated using an Ångstrom Engineering electron beam to evaporate alternating λ/4 layers of SiO_2_ and Nb_2_O_5_. The hybrid cavities had 1, 3, 4, 5, 6 or 10 pairs on top of a 200 nm thick Ag layer. The 10-pair bottom DBR in the DBR–DBR cavity used in the condensation measurements was fabricated by Helia Photonics Ltd.

### Transfer matrix reflectivity modelling

To model the Rabi splittings and linewidths, a multiple-peak Voigt profile was fit to the BODIPY-Br extinction coefficient (calculated from the UV–Vis absorption data) to describe the exciton. The Voigt function was chosen as it was found that using a simple Lorentz function was not sufficient to properly model the Rabi splittings and LPB linewidths of the cavities. The background refractive index of the BODIPY-Br/PS film was assumed to be similar to that of the polystyrene. The refractive indices for polystyrene and Ag were obtained from Refs.^43,44^, respectively through the Refractiveindex.info database.

### Basic optical characterisation

UV–Vis absorption measurements on BODIPY-Br films were carried out using a Horiba Fluoromax 4 fluorometer using a xenon lamp. Angle-dependent reflectivity measurements used a motorised goniometer to scan from 10° to 70° in 1° increments. An Ocean Optics Deuterium-Tungsten lamp (DH-2000-BAL) and an Andor Shamrock SR-303i-A CCD spectrometer were coupled by an optic fibre to the excitation and collection arms of the goniometer, respectively. In order to collect data on the LPB linewidths, *k*-space images were taken by focusing a pulsed, frequency-doubled Ti:sapphire (Coherent MIRA 900) laser at 445 nm onto the cavity at normal incidence using an Edmund Optics 20 × HR infinity corrected objective (numerical aperture = 0.6). A beamsplitter and lens were used to direct and focus the reflected light onto the CCD of the same Andor Shamrock spectrometer. This same setup was used for the PL mapping measurements with a 445 nm continuous wave ThorLabs diode laser used to excite the cavities. For these measurements, the sample was positioned on an (*x*, *y*) motorised stage which was used to move the sample in a raster pattern.

### Condensation measurements

A Ti:Sapphire laser (Coherent LibraHE) with ~ 150 fs pulses was tuned to the first Bragg minimum of each cavity (467 and 462 nm for the hybrid and DBR cavities, respectively) using an OPerA Solo optical parametric amplifier. This pump beam was focused onto the sample by a Mitutoyo Plan Apo 20× microscope objective (numerical aperture = 0.42) to produce a pump spot of FWHM diameter ~ 30 μm. The emission from the cavities was collected in reflection configuration by the same objective. To block the residual reflected light from the excitation beam a Semrock long-pass filter BLP01-473R-25 was placed in the collection path. The filtered photoluminescence was then coupled into a 750 mm focal length spectrometer (Princeton Instruments SP2750) equipped with an electron-multiplying CCD camera (Princeton Instruments ProEM-HS 1024 × 1024). A 300 grooves mm^−1^ grating and a 20 µm entrance slit were used to achieve a spectral resolution of 150 pm.

### AFM, SEM and profilometry measurements

AFM measurements were performed in tapping mode in air using a Dimension 3100 (Veeco) scanning probe microscope equipped with a nanoscope 3A feedback controller. The AFM tips were silicon Scout 350 RAI (NuNano) probes with a spring constant of 42 N/m and resonance frequency of 350 kHz. Data was levelled by mean plane subtraction using Gwyddion 2.55 software and roughness values were extracted using the Gwyddion statistical quantities tool. Scans were in most cases (10 × 10) μm^2^, but some were (2 × 2) μm^2^ or (1 × 1) μm^2^. The size of the scan did not seem to have an effect on the RMS roughness values determined. SEM images were taken using an FEI NovaNano SEM operating at a beam energy of 1.5 kV at a working distance of 4–5 mm, with an in-lens detector used to collect backscattered electrons. Samples for cross-sectional imaging were first scribed using a diamond stylus and then snapped. For the profilometry measurements, the same Bruker DektakXT profilometer as described above was used to make (100 × 100) μm^2^ surface maps.

## Supplementary Information

Supplementary Information.
