# Supplementary material for: Polariton condensation in an organic microcavity utilising a hybrid metal-DBR mirror
Source: Sci Rep. 2021 Oct 22;11:20879. doi: 10.1038/s41598-021-00203-y (PMC8536762; doi:10.1038/s41598-021-00203-y)
Supplement: Supplementary file 1 — Supplementary Information. [file 41598_2021_203_MOESM1_ESM.pdf]

# Supplementary Information: Polariton condensation in an organic microcavity utilising a hybrid metal-DBR mirror

Kirsty E. McGhee[1], Anton Putintsev[2], Rahul Jayaprakash[1], Kyriacos Georgiou[1,3], Mary E. O'Kane[1], Rachel C. Kilbride[1], Elena J. Cassella[1], Marco Cavazzini[4], Denis A. Sannikov[2], Pavlos G. Lagoudakis[2,5], and David G. Lidzey[1]*

1. Department of Physics and Astronomy, University of Sheffield, Hicks Building, Hounsfield Road, Sheffield S3 7RH, U.K.
2. Centre of Photonics and Quantum Materials, Skolkovo Institute of Science and Technology, Moscow, Russian Federation, 121205
3. Department of Physics, University of Cyprus, P.O. Box 20537, Nicosia 1678, Cyprus
4. Consiglio Nazionale delle Ricerche, Istituto di Scienze e Tecnologie Chimiche "Giulio Natta", Via C. Golgi 19, 20133 Milano, Italy
5. Department of Physics and Astronomy, University of Southampton, University Road, Southampton SO17 1BJ, U.K.

* Corresponding author: d.g.lidzey@sheffield.ac.uk

## Contents:

**1.** Silver mirror reflectivity

**2.** Electric field simulations

**3.** Polariton condensation

**4.** PL mapping

**5.** AFM Imaging

**6.** Surface Profilometry imaging

## 1. Silver mirror reflectivity

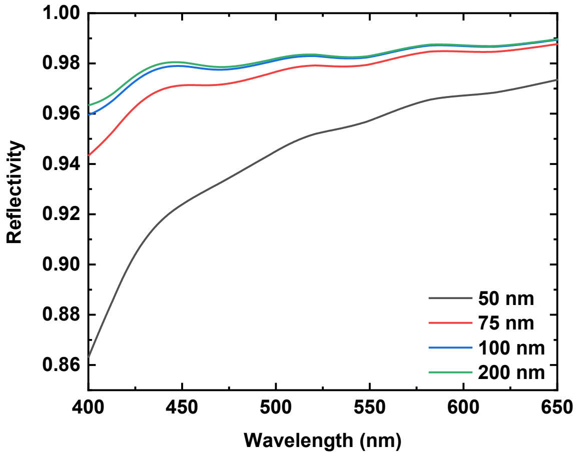


Figure S1. TMR model reflectivity of different thickness silver films. This indicates as expected that the reflectivity increases as thickness increases. Our model indicates that reflectivity does not increase once thickness exceeds ~ 200 nm and thus this is the thickness of silver used in our experiments.

## 2. Electric field simulations

In the main text, we show that the hybrid mirror cavities have enhanced Rabi splittings compared to the DBR-DBR cavity, which we attribute to the shorter effective length ($L_{eff}$) in cavities containing a layer of Ag. In the cavities studied here there are unintended thickness variations of the active layer, and thus the effective cavity lengths do not increase monotonically with increasing number of DBR pairs in the hybrid mirror. This is shown in Figure S2(a), where it can be seen that the 5-pair hybrid cavity has the longest effective length. In Figure S2(b), we plot $L_{eff}$ assuming a constant active layer thickness of 200 nm. It can be seen from this that $L_{eff}$ increases as the number of DBR pairs that are added to the hybrid mirror increases. Furthermore, we find as expected that the 10-pair hybrid and DBR-DBR cavities have the longest $L_{eff}$. The large $L_{eff}$ of the 5-pair hybrid cavity shown in part (a) therefore principally results from an increased active layer thickness. This effect is also evident in Figure 3(c) (see main paper), where the reduced cavity mode linewidth observed in the 5-pair hybrid cavity results from its increased photonic character. Because of this complicating effect of thickness variation, we simply plot the penetration depth into the bottom mirror in Figure 3(b) (main paper), rather than $L_{eff}$.

We note that the Rabi splitting in planar cavities is dependent on the number of absorbers per unit length. Hence, in cavities in which the only difference is the active layer thickness (and hence the polariton detuning), the Rabi splitting is expected to remain roughly constant. In our experiments therefore, the small unintended variations in active layer thickness are not expected to substantially change the Rabi splitting energy. Rather we attribute the observed variation in Rabi splitting in the different cavities to the different penetration depths of the optical field into the bottom mirror. Additionally, in Figure S3 we plot the electric field distribution of the cavity mode and the refractive index in the active layer and bottom mirror for the 1-pair and 5-pair hybrid cavities and the DBR-DBR control.

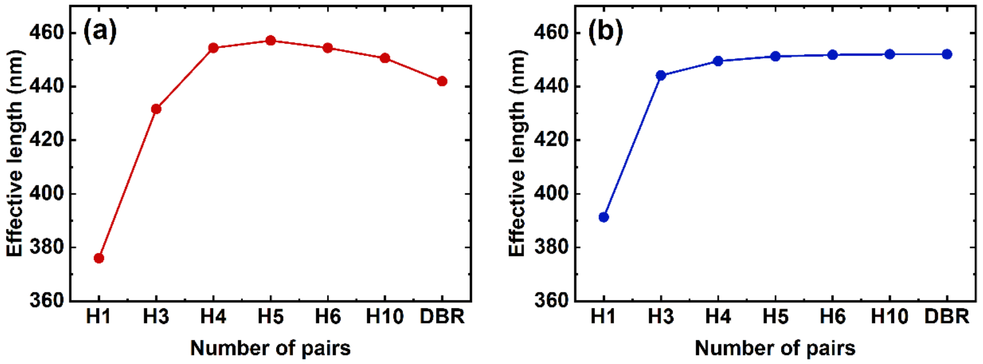


Figure S2. Effective lengths of the hybrid and DBR cavities. (a) shows the actual effective length, while (b) shows the effective length assuming a constant active layer thickness of 200 nm. From this it can be seen that the effective length increases as an increasing number of DBR pairs are added to the bottom mirror.

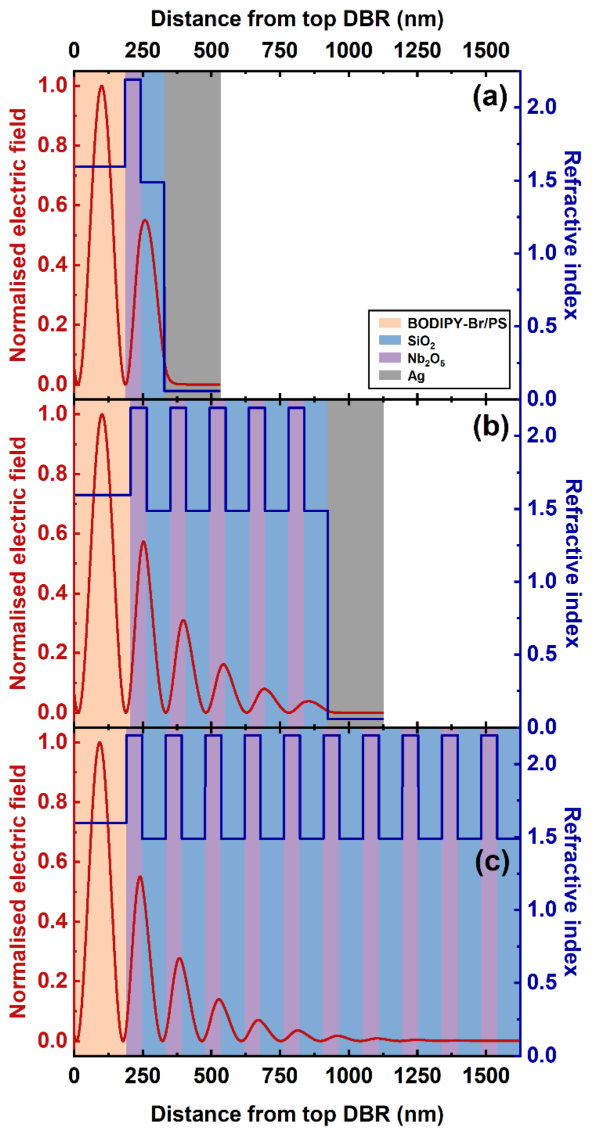


Figure S3. Electric field simulations of the cavity mode (red) and refractive index (blue) for (a) the 1-pair hybrid cavity, (b) the 5-pair hybrid cavity, and (c) the DBR-DBR control cavity. From this, it can be seen that the penetration length of the E-field changes in the different mirrors. Here, we omit the top DBR mirror as it is the same for all cavities, and thus the $x$-axis denotes the distance from the interface between the top DBR and the active layer. The different layers are shaded for clarity.

## 3. Polariton condensation

### 3.1 Blueshift

We find that the the blueshift that occurs in the hybrid cavity above threshold is twice that of the DBR cavity. We attempt to model this effect by predicting the blueshift of the LPB as we progressively reduce the oscillator strength of the BODIPY-Br active layer in the DBR-DBR and hybrid-DBR cavities (see Figure S4). It can be seen that in both cavities, we observe a linear shift of the bottom of the LPB as we start to saturate the excitonic layer, with this effect being greater in the hybrid cavity. This effect is likely explained by the increased Rabi splitting observed in the hybrid cavity. Indeed, we note that the energy difference between the bottom of the lower polariton branch and the minimum of the uncoupled photon mode ($k = 0$) is a direct function of the Rabi splitting energy. This suggests therefore that any given reduction in oscillator strength will generate a larger blueshift in cavities having a greater Rabi splitting energy, a result confirmed by our experiments. We note however that our TMR model suggests that the blueshift in the hybrid mirror cavity should be around 30% larger than the DBR-DBR cavity. Experimentally, however, we observe an energy shift in the hybrid mirror cavity which is a factor of two larger than in the DBR-DBR cavity. At present, we are not able to reconcile these observations. Further systematic experiments are planned to explore such effects in more detail.

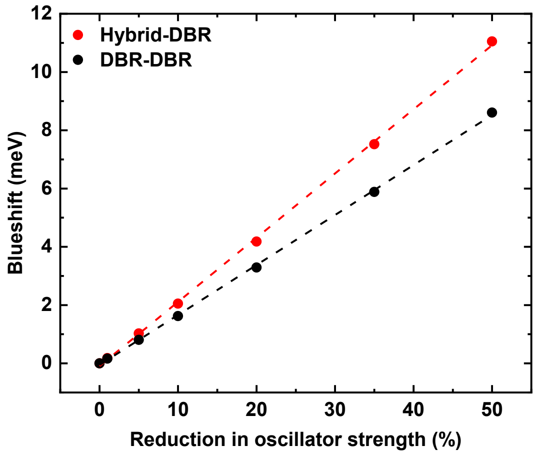


Figure S4. TMR simulations of the blueshift in the 1-pair hybrid (red) and DBR (black) cavities used in the condensation measurements as the oscillator strength of the BODIPY-Br/PS active layer is reduced.

### 3.2 Real-space condensate imaging

In Figure S5, we show the real-space images of the 1-pair hybrid and DBR cavities below and above threshold. It can clearly be seen that a very similar break-up of the condensate is seen in both the hybrid mirror cavity and the DBR cavity. This effect is ascribed to interactions between the condensate and the exciton reservoir.

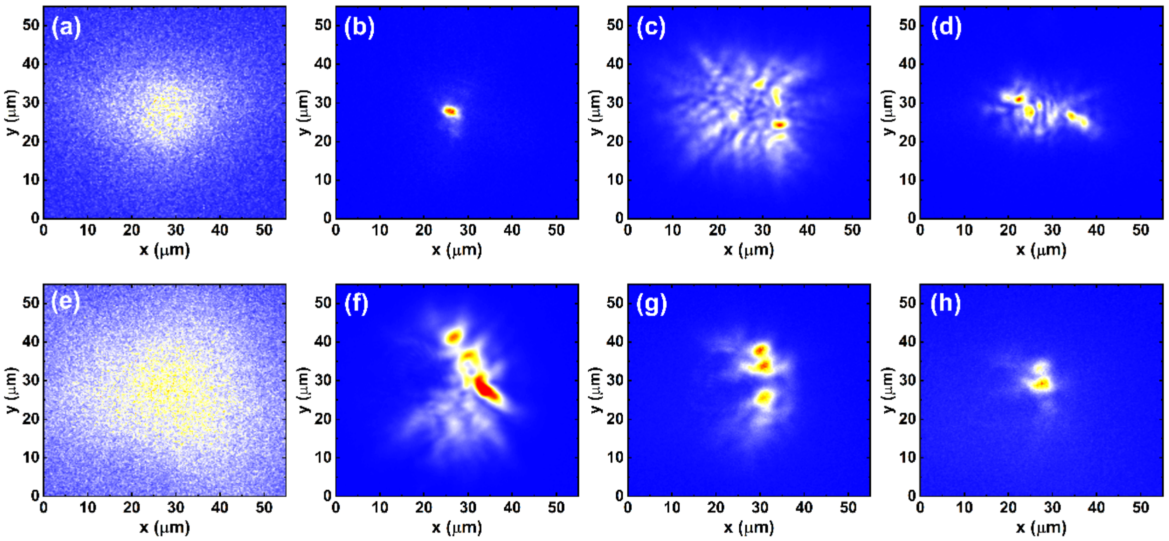


Figure S5. Part (a) shows a real space image of polariton luminescence emitted from the hybrid mirror cavity when pumped below threshold. Parts (b), (c) and (d) are images of polariton condensates generated at different regions in the hybrid mirror cavity when pumped above threshold. Part (e) shows a real space image of polariton luminescence emitted from the DBR-DBR control cavity when pumped below threshold. Parts (f), (g) and (h) are images of polariton condensates generated at different regions in the DBR-DBR control cavity when pumped above threshold. In all cases, the size of the image is (55 x 55) $\mu m^2$.

## 4. PL mapping below condensation threshold

In Figure S6(a), we show an additional sub-threshold PL map recorded for the DBR-DBR cavity, showing a similar trend in energy landscape to the map shown in Figure 6(a) of the main text. In part (b), we show the LPB spectra that correspond to the highest and lowest peak energies recorded in the map shown in Figure 6(a). In part (c) we show the 585 nm spectral line of a Ne/Ar lamp measured using the Andor spectrometer used in the PL mapping measurements. A Lorentz function was fitted to this spectrum and from this we determine a spectral resolution of 0.68 meV.

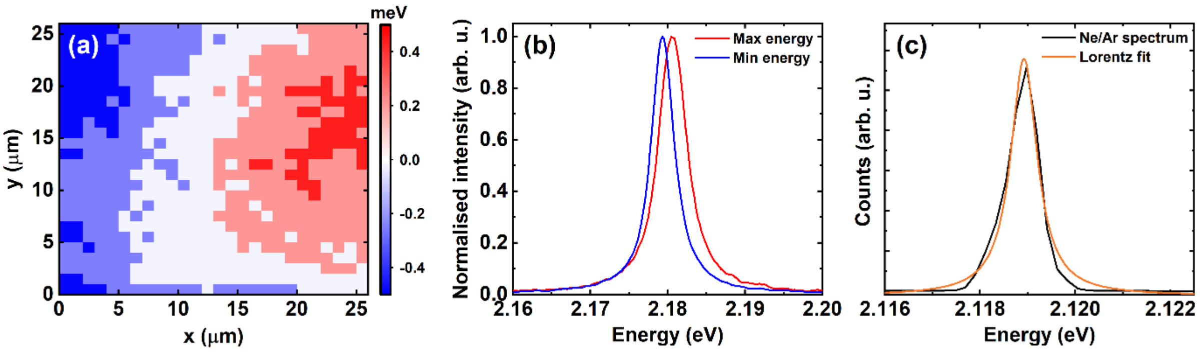


Figure S6. Additional photoluminescence map data. Part (a) shows an additional PL map for the DBR-DBR control cavity. Part (b) shows the LPB spectra corresponding to the maximum (red) and minimum (blue) peak energies from the map in Figure 6(a) of the main text. Part (c) shows the spectral line of a Ne/Ar lamp recorded using the PL mapping spectrometer (black) and a Lorentz fit to it (orange). From this, a spectral resolution of 0.68 meV is determined.

## 5. Atomic force microscopy (AFM)

To understand the origin of the enhanced energetic inhomogeneity in the hybrid mirror cavity, we have used AFM to determine whether the surface of the hybrid mirror is rougher than that of the DBR used in the DBR-DBR control cavity. AFM images were recorded on a series of surfaces as shown in Figure S7, including (a) the first Ag layer, (b) a SiO2 film on a glass surface, (c) a SiO2 film on a Ag film, and (d) a 1-pair hybrid mirror. This is compared with (e) a conventional 10-pair DBR mirror. The root mean square roughness from each surface is tabulated in Table 1 of the main manuscript. Each AFM scan is performed over an area of (10 x 10) $\mu m^2$. Our results indicate a general increase in surface roughness of the hybrid mirror compared to the DBR that SEM images indicate result from incomplete adhesion between the $SiO_2$ and the Ag film (see main text for details).

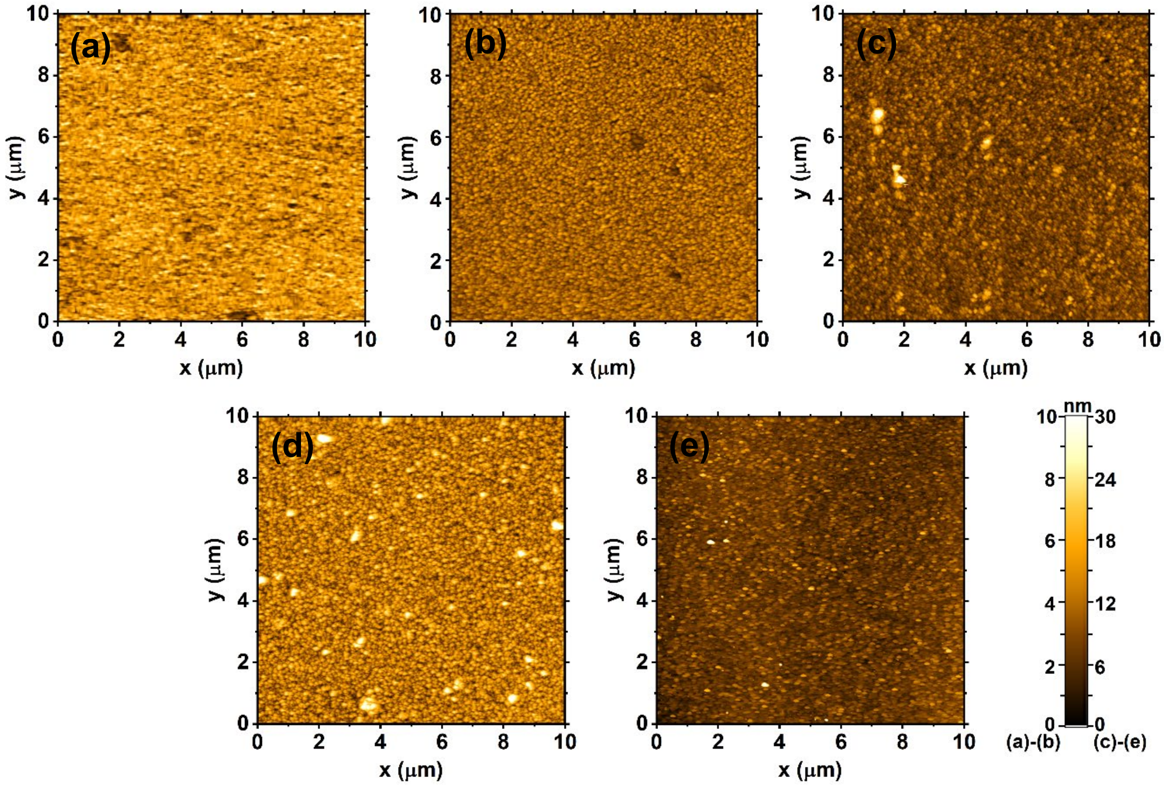


Figure S7. Atomic force microscopy maps of (a) Ag, (b) $SiO_2$, (c) Ag coated with $SiO_2$, (d) the 1-pair hybrid mirror ($Ag/SiO_2/Nb_2O_5$), and (e) the 10-pair DBR mirror. The colour scale in (a) and (b) corresponds to 10 nm, whereas for (c), (d) and (e) it corresponds to 30 nm. All structures were deposited on top of quartz-coated glass.

The effect of deposition rate of $SiO_2$ onto Ag was also investigated using AFM. It was found that the surface roughness was not significantly altered by changing the deposition rate of $SiO_2$ onto an Ag film, with RMS roughness values obtained of 2.91 nm, 2.91 nm and 2.98 nm for deposition rates of 0.5 Å/s, 1 Å/s and 2 Å/s, respectively

## 6. Surface profilometry imaging

In addition to the AFM mapping, the surface of the different structures were mapped over a larger area using a Bruker DektakXT profilometer. These images were taken over an area of (100 x 100) $\mu m^2$ and an example map for each structure is shown in Figure S8, with average roughness values given in Table S1. From the table, it can be seen that this data agrees with the AFM in that both the Ag and $SiO_2$ films are very smooth compared to the 1-pair hybrid mirror and the Ag/$SiO_2$ bilayer (although over such a large area, the actual roughness values are higher than for the AFM). This data also confirms that the 10-pair DBR is smoother than the 1-pair hybrid.

In the Ag/$SiO_2$ bilayer in Figure S8(c), it can be see that there is surface debris, and this results in a very high RMS roughness. We believe this may be caused by the Dektak tip penetrating the voids between the two layers (seen in the SEM images) as it moves across the sample surface. This then drags fragmented $SiO_2$ across the sample, creating such debris features. Figure S8(f) shows a microscope image (20x magnification), which shows the defects made in the film by the Dektak measurement. No such defects were found outside the measurement areas or in the other samples. We believe this is further evidence for the poor adhesion between $SiO_2$ and Ag.

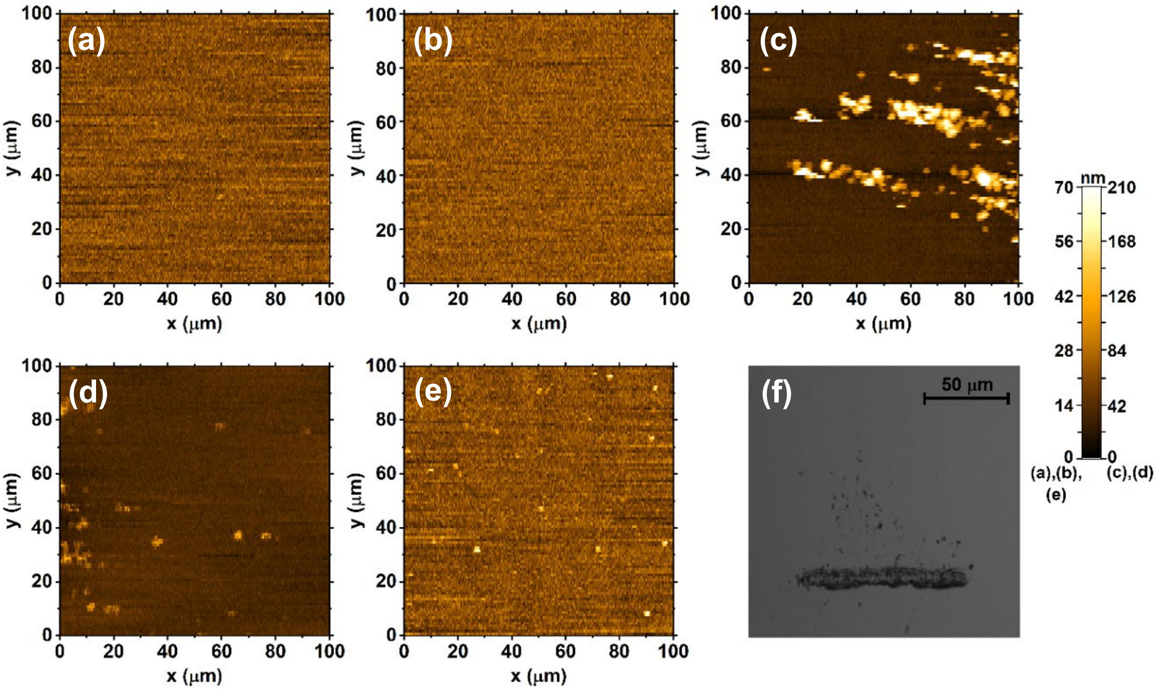


Figure S8. Surface profilometry (Dektak) maps of (a) Ag, (b) $SiO_2$, (c) Ag coated with $SiO_2$, (d) the 1-pair hybrid mirror (Ag/$SiO_2$/$Nb_2O_5$), and (e) the 10-pair DBR mirror. The colour scale in (a), (b) and (e) covers 70 nm, whereas for (c) and (d) it covers 210 nm. All structures were deposited on top of quartz-coated glass. Part (f) is a microscope image (20x magnification) of the Ag/$SiO_2$ film after performing Dektak measurements. The thick dark line is an artefact caused by the initial contact from the Dektak tip and indicates the start of the measurement area. This line is apparent on all samples; however, the dark specks above it are only seen in the Ag/$SiO_2$ image and only after the measurement. We believe they are fragments of $SiO_2$ dragged across the film by the tip due to its poor adhesion to the Ag.

| Structure | Ag | $SiO_2$ | Ag + $SiO_2$ | 1-pair hybrid | 10-pair DBR |
|---|---|---|---|---|---|
| RMS roughness (nm) | 4.6 | 5.3 | 33.5 | 14.4 | 5.6 |

Schematic

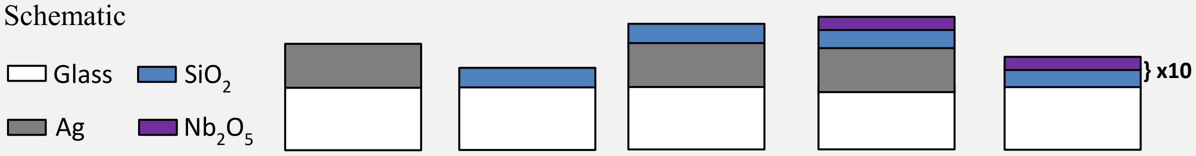


Table S1. Root mean square (RMS) roughness of the different structures investigated, with a schematic of the structure shown beneath. These values were obtained by averaging multiple Dektak maps.
